# Immune Cell Infiltration of the Primary Tumor Microenvironment Predicted the Treatment Outcome of Chemotherapy With or Without Bevacizumab in Metastatic Colorectal Cancer Patients

**DOI:** 10.3389/fonc.2020.581051

**Published:** 2021-01-27

**Authors:** Yixing Wang, Jun Dong, Qi Quan, Shousheng Liu, Xiuxing Chen, Xiuyu Cai, Huijuan Qiu, Bei Zhang, Guifang Guo

**Affiliations:** ^1^VIP Department, Sun Yat-sen University Cancer Center, Guangzhou, China; ^2^State Key Laboratory of Oncology in South China, Sun Yat-sen University Cancer Center, Guangzhou, China; ^3^Collaborative Innovation Center for Cancer Medicine, Sun Yat-sen University Cancer Center, Guangzhou, China; ^4^Department of Medical Oncology, Sun Yat-sen University Cancer Center, Guangzhou, China

**Keywords:** tumor microenvironment, immune cells, metastases colorectal cancer, treatment outcome, bevacizumab

## Abstract

**Background:**

With the interest in cancer immunotherapy, it may be possible to combine immunotherapy with bevacizumab and chemotherapy. We evaluated whether tumor-infiltrating immune cells are associated with the efficacy of chemotherapy with or without bevacizumab for the treatment of metastatic colorectal cancer (mCRC).

**Methods:**

This study enrolled mCRC patients on standard treatment with available detailed data and tumor tissue at Sun Yat-sen University Cancer Center between July 1, 2005, and October 1, 2017. CD3+ and CD8+ T cell densities examined by immunohistochemistry in both the tumor core (CT) and invasive margin (IM) were summed as the Immunoscore, and the CD8+/CD3+ T cell ratio was calculated. The predictive and prognostic efficacies of tumor-infiltrating immune cells for progression-free survival (PFS) and overall survival (OS) were analyzed with Kaplan-Meier and Cox analyses.

**Results:**

The CD8+/CD3+ T cell ratio in the microenvironment was an independent prognostic factor for OS (28.12 mo vs. 16.56 mo, *P* = 0.017) among the 108 studied patients. In the chemotherapy only group, patients with a high Immunoscore had a high overall response rate (ORR, 40.0% vs. 60.0%, *P* = 0.022), those with a low CD8+/CD3+ T cell ratio in the microenvironment had a significantly longer PFS (8.64 mo vs. 6.01 mo, *P* = 0.017), and those with a high CD3+ T cell density in the CT had a longer OS (16.56 mo vs. 25.66 mo, *P* = 0.029). In the chemotherapy combined with bevacizumab group, patients with a higher CD8+ T cell density in the IM had a longer PFS (7.62 mo vs. 11.66 mo, *P* = 0.034) and OS (14.55 mo vs. 23.72 mo, *P* = 0.033).

**Conclusion:**

Immune cells in primary tumors play an important role in predicting mCRC treatment efficacy. CD8 predicts the effect of bevacizumab plus chemotherapy, while CD3 and CD8/CD3 predict chemotherapy efficacy.

## Introduction

Colorectal cancer (CRC) is the third most common cancer in the world. Metastases are present in approximately 25% to 30% of patients at diagnosis and develop in up to 50% of patients thereafter (1). Although advances have been made in the past 15 years, the 5-year survival rate of remains as low as approximately 20% (2–4). According to the American Joint Committee on Cancer (AJCC) and Union for International Cancer Control (UICC) TNM classification system, the prognosis of patients is predicted based on histopathological criteria of tumor invasion (5). There has been increasing attention has been paid to predicting CRC prognosis with a focus on tumor cells, mutation status, molecular pathways and immune cell infiltration (6).

To date, immunotherapeutics, against the programmed cell death protein-1 (PD-1)/programmed cell death protein-1 ligand (PD-L1) axis, have been approved by the Food and Drug Administration for metastatic colorectal cancer (mCRC) with microsatellite instability (MSI)-high (H) only (7). However, a minority (5%) of mCRC tumors show MSI and the vast majority of mCRC tumors have shown limited responses to these checkpoint inhibitors. In the new era of precision medicine, it is critical to identify predictive biomarkers to classify patients into different groups to receive different therapeutic regimens. The host immune response has been found to play an important role in determining the outcome of patients with CRC. Tumor-infiltrating lymphocytes (TILs) represent a host immune response that directly correlates with microinvasive status (8). In the context of cancer, T cells are often inactive or are a minimal proportion of the immune infiltrate (9). The elegant studies by Camus (10), Galon (11) and Pages (12) showed that the presence of immune infiltrate in primary CRC is an excellent positive prognostic indicator. It has been suggested that the analysis of immune cells in the microenvironment in combination with the AJCC/UICC stage could lead to a better determination of patient prognosis (13). In this study, we investigated CD3+ (total) and CD8+ (cytotoxic) T cells to determine the prognosis of mCRC patients on standard palliative treatment.

The current front-line treatment for mCRC patients is often a combination of chemotherapies and biotherapies, the anti-VEGF mAb and anti-EGFR mAb antiangiogenic agents. In the case of bevacizumab, VEGF in the tumor microenvironment drives angiogenesis and contributes to local immune evasion by the tumor (14). Clinical trials have confirmed that regorafenib, a potent inhibitor of angiogenic and oncogenic kinases, in combination of PD1 has encouraging antitumor activity in microsatellite stable (MSS) mCRC patients (15). It will be necessary to interrogate whether chemotherapy combined with bevacizumab is deleterious to the immune system in CRC patients, is immune inert or enhances certain immune components.

In the study reported herein, we analyzed whether CD3+ and CD8+ T cells in the tumor microenvironment influence the response to chemotherapy alone and in combination with bevacizumab. We hope to provide a rationale for the combined use of immunotherapeutics such as vaccines, immunomodulators such as immunocytokines, and mAbs against checkpoint inhibitors with chemotherapy and bevacizumab for the treatment of patients with mCRC.

## Materials and Methods

### Study Population

The study population comprised patients diagnosed mCRC at Sun Yat-sen University Cancer Center in China between July 1, 2005, and October 1, 2017, 2017. All patients had histologically proven CRC at the primary tumor site, and the pathology of all cases were adenocarcinomas and the tissues of primary cancer were available. At least four cycles of palliative chemotherapy were given. Moreover, patients had not previously received immunosuppressive therapy or anti-inflammatory medicine, such as recent exposure to steroids, or did not have a chronic inflammatory disease. This study was approved by the Institutional Review Board and Ethics Committee at Sun Yat-sen University Cancer Center.

### Treatment Protocols

The administration of chemotherapy was determined according to National Comprehensive Cancer Network guidelines by the physicians of Sun Yat-sen University Cancer Center. The chemotherapy regimens administered in this study included FOLFOX (oxaliplatin, 5-FU and leucovorin), FOLFIRI (irinotecan, 5-FU and leucovorin), or the above chemotherapy combined with target drug of bevacizumab or cetuximab.

### Data Collection

The patients were followed-up until July 2019 by hospital records. Our primary study endpoints were first-line progression-free survival (PFS), which was defined as the time from the initial palliative therapy to tumor progression, death from any cause, or the last follow-up before the initiation of second-line therapy, and overall survival (OS), which was defined as the time from the date of the first cycle of front-line therapy to the date of death from any cause. In addition, the objective response rate (ORR) and disease response rate (DCR) to the first-line treatment were also studied.

### Tissue Sample

All CRC tissues were surgical specimens which were taken from primary tumors of colorectal. All surgery samples were obtained before chemotherapy. All tissues were acquired from the sample bank of the pathology department.

### Immunohistochemical Staining

Paraffin-embedded slides were stained with monoclonal antibodies against CD3 and CD8 (Cell Signaling Technology, United States; Catalog No. 85016S and 85336S, respectively). Olympus digital slide scanners were used to scan stained sections from representative areas, and two independent pathologists, blinded to patient clinical information, took part in the recognition of the location of the core of the tumor (CT) and invasive margin (IM). ImageJ software (National Institutes of Health, Bethesda, MD, USA) was used for computer-aided calculations of the density of CD8+ and CD3+ T cells. The Immunoscore was evaluated according to the ways reported by Galon et al. (11). The assessment was based on the densities of CD8+ and CD3+ T cells with a cut-off of the median of each index, including CD8+ T cells in the CT and IM, CD3+ T cells in the CT and IM. And densities below the median were classified as low expression, those above the median were categorized as high expression. A high value was scored as 1, and low value was scored as 0. The sum of the scores of all indexes was calculated to determine the final Immunoscore. Immunoscores > 2 were defined as a high Immunoscore, while Immunoscores ≤ 2 were defined as a low Immunoscore. Furthermore, we calculated the CD8+/CD3+ T cell ratio in tumor center, IM, and the whole microenvironment.

### Statistical Analysis

Statistical analysis was performed with SPSS 24.0 for Windows (SPSS, Chicago, IL, USA). The associations between the expression of CD3+ T cells in either tumor center or invasive margin, CD8+ T cells in either tumor center or invasive margin, CD8/CD3, Immunoscore, and clinicopathological characteristics were assessed using the chi-square test or Fisher’s exact test, as appropriate. The comparison of densities of CD3 and CD8 according to the CT and IM in all 107 patients using the paired T-test. The Kaplan-Meier method and the log-rank test were used to investigate and compare prognostic roles in predicting PFS and OS. The COX multivariate analyses were used to determine the probability of clinical benefit, factors with potential prognostic significance in univariate analysis were included in multivariate analysis (*P*<0.600). All *P* values are two tailed. *P* < 0.05 indicated a significant difference.

## Results

### Patient Characteristics and Treatment

Initially, 1,307 mCRC patients were identified in the clinical database of our center, but only 292 had detailed data and well-preserved tumor specimens. Finally, 108 patients treated with standard palliative chemotherapy and efficacy evaluations were enrolled in our study. The basic characteristics of all the studied patients are shown in **Table 1**: the cohort included 68 males and 40 females aged 21 to 82 years, with a median age of 60 years. According to the splenic flexure of the colon, the primary tumor location was characterized as the right (28 patients) or left colon (80 patients). The pathological differentiation was identified as moderate in more than half of the tumors (65, 60.2%), as low in 41, and as high in only 2. The TNM stage was determined by the eighth AJCC standard before first-line palliative chemotherapy. No patient was diagnosed at T1, 2 patients were at T2, 64 were at T3, and 35 were at T4. The patients were almost equally distributed among different N stages, with 21 patients in N0, 36 in N1, and 38 in N2. Synchronous and metachronous metastases were present in 25 and 83 patients, respectively. Many factors associated with treatment choice and prognosis were also included in our study. Microsatellite status was available for 68 patients, and only 4 exhibited MSI. KRAS status was determined in 54 patients; 31 harbored wild-type KRAS, and 23 harbored mutated KRAS. NRAS and HRAS were shown to be wild type in the 29 and 28 evaluated patients, respectively, and BRAF was wild-type in all 37 evaluated patients. All 108 patients were treated with palliative therapy. Among them, 55 received FOLFOX/FOLFIRI alone as first-line treatment, 38 received bevacizumab plus FOLFOX/FOLFIRI, and 15 received cetuximab plus FOLFOX/FOLFIRI.

**Table 1:** Basic clinicopathological molecular characteristics of 108 metastasis colorectal cancer patients.

Variable		No. of patients (%)	Variable		No. of patients (%)
Sex	Male	68 (63.0%)	MS	MSS	64 (59.3%)
	Female	40 (37.0%)		MSI	4 (3.7%)
Age	<70	78 (72.2%)		NA	40 (37.0%)
	≥ 70	30 (27.8%)	KRAS	Wild-type	31 (28.7%)
Location	Right	28 (25.9%)		Mutation-type	23 (21.3%)
	Left	80 (74.1%)		NA	54 (50.0%)
Pathological differentiation	Poor	41 (38.0%)	NRAS	Wild-type	29 (26.9%)
	Moderate	65 (60.2%)		Mutation-type	0 (0.0%)
	Well	2 (1.9%)		NA	79 (73.1%)
T stage	T2	3 (2.8%)	HRAS	Wild-type	28 (25.9%)
	T3	64 (59.3%)		Mutation-type	0 (0.0%)
	T4	35 (32.3%)		NA	80 (74.1%)
	NA	6 (5.6%)	BRAF	Wild-type	37 (34.3%)
N stage	N0	21 (19.4%)		Mutation-type	0 (0.0%)
	N1	36 (33.3%)		NA	71 (65.7%)
	N2	38 (35.2%)	First-line chemotherapy	Cetuximab +FOLFOX/FOLRIRI	15 (13.9%)
	NA	13 (12.0%)		Bevacizumab + FOLFOX/FOLRIRI	38 (35.2%)
Synchronous/metachronous metastasis	Synchronous metastasis	25 (23.1%)		FOLFOX/FOLRIRI	55 (50.9%)
	Metachronous metastasis	83 (76.9%)			

Location: The primary tumor location was classified as right-sided or left-sided according to the splenic flexure. NA, not applicable.

### Assessment of Immune Cell Infiltration

**Figure 1:**
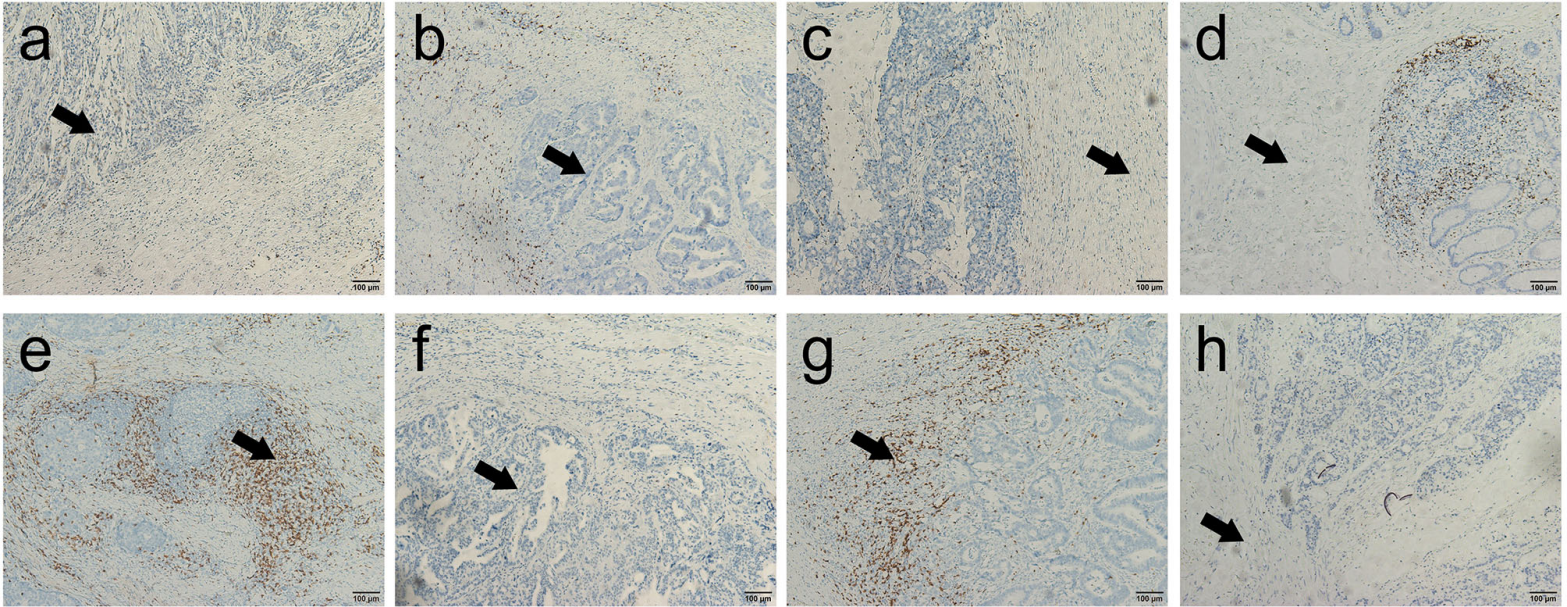
Representative immunohistochemical images of CD3+ and CD8+ T cells in the core of the tumor (CT) and in the invasive margin (IM) of colorectal cancer (200×). **(A, B)** Representative images of high-density and low-density CD3+ T cells in the center of the colorectal cancer; **(C, D)** Representative images of high-density and low-density CD3+ T cells in the invasive margin of the colorectal cancer; **(E, F)** Representative images of high-density and low-density CD8+ T cells in the center of the colorectal cancer; **(G, H)** Representative images of high-density and low-density CD8+ T cells in the invasive margin of the colorectal cancer.

We assessed immune cell infiltration (CD3 and CD8) in specimens from primary colorectal tumors. CD8+ T cell expression data in tissue was available for 108 patients. Immunohistochemical staining of CD3 was unsuccessful for one patient, so 107 patients had CD3+ T cell expression, CD8+/CD3+ T cell expression and Immunoscore data. The median density of CD3+ T cells in the tumor core (CT) and invasive margin (IM) was 1,048/mm^2^ (8/mm^2^–11,917/mm^2^) and 1,177/mm^2^ (33/mm^2^–11,551/mm^2^), respectively, and the corresponding values for CD8+ T cells were 105/mm^2^ (2/mm^2^–4,178/mm^2^) and 231/mm^2^ (1/mm^2^–2,705/mm^2^), respectively. The median ratio of CD8+ to CD3+ T cells in the CT, IM and total microenvironment was 0.12, 0.13, and 0.11, respectively. Low expression was defined as a value below the median, and high expression was defined as a value above the median (**Figure 1**). The correlation between CD3+ expression and CD8+ expression was significantly associated both in IM (*P*=0.000) and CT (*P*=0.000). The CD3+ expression was significantly higher in IM than in CT (*P*=0.000), But CD8+ expression did not show the trend (*P*=0.062).

### Association Between Basic Characteristics and Immune Cells in the Tumor Microenvironment

The percentage of CD3+ T cells in both the CT (66.7% vs. 33.3%, *P* = 0.033) and IM (73.3% vs. 26.7%, *P* = 0.003) was lower in older patients, as was the Immunoscore (90.0% vs. 10.0%, *P* = 0.001). The ratio of CD8+ to CD3+ T cells in the IM (30.0% vs. 70.0%, *P* = 0.017) and the total microenvironment (30.0% vs. 70.0%, *P* = 0.019) was higher in older patients. CD8+ expression in the IM was lower in patients with N0 stage disease (33.3% vs. 66.7%, *P* = 0.045). Patients with synchronous metastasis had a lower percentage of CD3+ T cells in both the CT (68.0% vs. 32.0%, *P* = 0.042) and IM (76.0% vs. 24.0%, *P* = 0.03) and had a higher ratio of CD8+ to CD3+ T cells in the CT (28.0% vs. 72.0%, *P* = 0.023), IM (24.0% vs. 76.0%, *P* = 0.005), and total microenvironment (16.0% vs. 84.0%, *P* = 0.000). We did not find any relationship between microsatellite status and the expression of immune cells in the CT or IM, the ratio of CD8+ to CD3+ T cells, or the Immunoscore; the same was true for KRAS status. The detailed results are shown in **Table 2**.

**Table 2:** Correlation between basic characteristics and immune cells in the microenvironment.

Variable	N=108	CD3+ T cells in the CT				CD3+ T cells in the IM				CD8+ T cells in the CT			CD8+ T cells in the IM			CD8+/CD3+ T cells in the CT				CD8+/CD3+ T cells in the IM				CD8+/CD3+ T cells in the microenvironment					Immunoscore			
		Low	High	NA	*P*	Low	High	NA	*P*	Low	High	*P*	Low	High	*P*	Low	High	NA	*P*	Low	High	NA	*P*	Low	High	NA		*P*	Low	High	NA	*P*
Sex					0.428				0.692			0.842			0.550				0.690				1.000				0.844					0.835
Male	68	31 (0.463)	36 (0.537)	1		32 (0.478)	35 (0.522)	1		35 (0.515)	33 (0.485)		36 (0.529)	32 (0.421)		34 (0.507)	33 (0.493)	1		33 (0.493)	34 (0.507)	1		32 (0.478)	35 (0.522)	1			45 (0.672)	22 (0.328)	1	
Female	40	22 (0.550)	18 (0.450)			21 (0.525)	19 (0.475)			19 (0.475)	21 (0.525)		18 (0.450)	32 (0.550)		18 (0.450)	22 (0.550)			20 (0.500)	20 (0.500)			20 (0.500)	20 (0.500)				26 (0.650)	14 (0.350)		
Age					0.033				0.003			1.000			1.000				0.137				0.017				0.019					0.001
<70	78	33 (0.429)	44 (0.571)	1		31 (0.408)	46 (0.592)	1		39 (0.500)	39 (0.500)		39 (0.500)	39 (0.500)		41 (0.532)	36 (0.468)	1		44 (0.571)	33 (0.429)	1		43 (0.558)	34 (0.442)	1			44 (0.571)	33 (0.429)	1	
≥70	30	20 (0.667)	10 (0.333)			22 (0.733)	8 (0.267)			15 (0.500)	15 (0.500)		15 (0.500)	15 (0.500)		11 (0.367)	19 (0.633)			9 (0.300)	21 (0.700)			9 (0.300)	21 (0.700)				27 (0.900)	3 (0.100)		
Location					1.000				0.385			0.511			0.123				1.000				1.000				0.829					0.643
Right	28	14 (0.500)	14 (0.500)			16 (0.571)	12 (0.429)			16 (0.571)	12 (0.429)		18 (0.643)	10 (0.357)		14 (0.500)	14 (0.500)			14 (0.500)	14 (0.500)			13 (0.464)	15 (0.536)				20 (0.714)	8 (0.286)		
Left	80	39 (0.494)	40 (0.506)	1		37 (0.468)	42 (0.532)	1		38 (0.475)	42 (0.525)		36 (0.450)	44 (0.550)		38 (0.481)	41 (0.519)	1		39 (0.494)	40 (0.506)	1		39 (0.494)	40 (0.506)	1			51 (0.646)	28 (0.354	1	
Pathological differentiation					0.428				0.844			0.234			0.234				1.000				0.428				0.843					0.403
Poor	41	18 (0.493)	23 (0.561)			21 (0.512)	20 (0.488)			17 (0.415)	24 (0.585)		17 (0.415)	24 (0.585)		20 (0.485)	21 (0.512)			18 (0.439)	23 (0.561)			19 (0.463)	22 (0.537)				25 (0.610)	16 (0.390)		
Moderate and well	67	35 (0.530)	31 (0.470)	1		32 (0.485)	34 (0.515)	1		37 (0.552)	30 (0.448)		37 (0.552)	30 (0.448)		32 (0.485)	34 (0.515)	1		35 (0.530)	31 (0.470)	1		33 (0.500)	33 (0.500)	1			46 (0.697)	20 (0.303)	1	
T stage					0.531				0.059			0.409			0.214				0.677				0.535				0.094					0.510
T1+T2+T3	67	32 (0.485)	34 (0.515)	1		29 (0.439)	37 (0.561)	1		35 (0.522)	32 (0.478)		29 (0.433)	38 (0.567)		32 (0.485)	34 (0.515)	1		33 (0.500)	33 (0.500)	1		35 (0.530)	31 (0.470)	1			42 (0.636)	24 (0.364)	1	
T4	35	20 (0.571)	15 (0.429)			23 (0.657)	12 (0.343)			15 (0.429)	20 (0.571)		20 (0.571)	15 (0.429)		15 (0.429)	20 (0.571)			15 (0.429)	20 (0.571)			12 (0.343)	23 (0.657)				25 (0.714)	10 (0.286)		
NA	6																															
N stage					0.213				1.000			1.000			0.045				0.614				0.321				0.321					0.793
No	21	8 (0.400)	12 (0.600)	1		11 (0.550)	9(0.450)	1		10 (0.476)	11 (0.524)		14 (0.667)	7 (0.333)		10 (0.500)	10 (0.500)	1		11 (0.550)	9 (0.450)	1		11 (0.550)	9 (0.450)	1			14 (0.700)	6 (0.300)	1	
N1+N2	74	42 (0.568)	32 (0.432)			40 (0.541)	34 (0.459)			35 (0.473)	39 (0.527)		29 (0.392)	45 (0.608)		31 (0.419)	43 (0.581)			31 (0.419)	43 (0.581)			31 (0.419)	43 (0.581)				48 (0.649)	26 (0.351)		
NA	13																															
M stage					0.042				0.003			1.000			1.000				0.023				0.005				0.000					0.146
Synchronous metastasis	25	17 (0.680)	8 (0.320)			19 (0.760)	6 (0.240)			13 (0.520)	12 (0.480)		13 (0.520)	12 (0.480)		7 (0.280)	18 (0.720)			6 (0.240)	19 (0.760)			4 (0.160)	21 (0.840)				20 (0.800)	5 (0.200)		
Metachronous metastasis	83	36 (0.439)	46 (0.561)	1		34 (0.415)	48 (0.585)	1		41 (0.494)	42 (0.506)		41 (0.494)	42 (0.506)		45 (0.549)	37 (0.451)	1		47 (0.573)	35 (0.427)	1		48 (0.583)	34 (0.415)	1			51 (0.622)	31 (0.378)	1	
MS					1.000				1.000			0.614			1.000				0.356				0.342				0.329					0.652
MSS	64	30 (0.476)	33 (0.524)	1		29 (0.460)	34 (0.540)	1		33 (0.516)	31 (0.484)		31 (0.484)	33 (0.516)		33 (0.524)	30 (0.476)	1		34 (0.540)	29 (0.460)	1		35 (0.556)	28 (0.444)	1			38 (0.603)	25 (0.397)	1	
MSI	4	2 (0.500)	2 (0.500)			2 (0.500)	2 (0.500)			1 (0.250)	3 (0.750)		2 (0.500)	2 (0.500)		1 (0.250)	3 (0.750)			1 (0.250)	3 (0.750)			1 (0.250)	3 (0.750)				3 (0.750)	1 (0.250)		
NA	40																															
KRAS					0.256				0.371			0.107			0.787				0.256				0.773				1.000					0.401
W	31	9 (0.290)	22 (0.710)			8 (0.258)	23 (0.742)			13 (0.419)	18 (0.581)		16 (0.516)	15 (0.484)		22 (0.710)	9 (0.290)			19 (0.613)	12 (0.387)			20 (0.645)	11 (0.355)				15 (0.484)	16 (0.516)		
M	23	10 (0.455)	12 (0.545)	1		9 (0.409)	13 (0.591)	1		15 (0.652)	8 (0.348)		13 (0.565)	10 (0.435)		12 (0.545)	10 (0.455)	1		15 (0.682)	7 (0.318)	1		14 (0.636)	8 (0.364)	1			14 (0.636)	8 (0.364)	1	
NA	54																															

Location: The primary tumor location was classified as right-sided or left-sided according to the splenic flexure. CT: Core of the tumor; IM: Invasive margin; NA, not applicable.

### Prognostic Value of Immune Cell Presence in All 108 Enrolled Patients

The median OS of all patients, regardless of chemotherapy regimen, was 21.8 mo (3.0 mo - 60.9 mo). Synchronous/metachronous metastasis (14.55 mo vs. 23.72 mo, *P* = 0.006) and the ratio of CD8+ to CD3+ T cells in the microenvironment (28.12 mo vs. 16.56 mo, *P* = 0.017) were prognostic factors for OS. The most common factors potentially affecting OS were analyzed with Cox analysis as shown in **Table 3**. None of the factors influenced progression-free survival (PFS), while synchronous or metachronous metastasis was the only variable associated with OS.

**Table 3:** Univariate and multivariate analyses of the influence of immune cell infiltration and clinicopathological factors on survival in all enrolled patients of 108.

	PFS				OS			
	Univariate analysis		Multivariate analysis		Univariate analysis		Multivariate analysis	
	HR (95% CI)	*P*	HR (95% CI)	*P*	HR (95% CI)	*P*	HR (95% CI)	*P*
Age	0.997 (0.613, 1.621)	**0.990**			0.816 (0.478, 1.394)	**0.456**		
Tumor location	1.217 (0.756, 1.959)	**0.420**			1.038 (0.578, 1.836)	**0.897**		
Pathological grade	1.292 (0.834, 2.002)	**0.251**	1.549 (0.962, 2.494)	**0.072**	1.358 (0.819, 2.252)	**0.236**		
T stage	0.820 (0.520, 1.293)	**0.393**			1.097 (0.646, 1.862)	**0.732**		
N stage	0.937 (0.556, 1.581)	**0.808**			1.008 (0.519, 1.959)	**0.982**		
Synchronous/metachronous metastasis	1.071 (0.628, 1.827)	**0.801**			0.455 (0.257, 0.805)	**0.007**	0.488 (0.267, 0.891)	**0.019**
CD3+ T cells in the CT	0.774 (0.506, 1.184)	**0.237**			0.615 (0.367, 1.029)	**0.007**		
CD3+ T cells in the IM	0.841 (0.549, 1.290)	**0.428**			0.765 (0.458, 1.277)	**0.305**		
CD8+ T cells in the CT	1.065 (0.699, 1.624)	**0.769**			1.108 (0.677, 1.811)	**0.683**		
CD8+ T cells in the IM	0.822 (0.533, 1.270)	**0.378**			0.953 (0.581, 1.564)	**0.850**		
CD8+/CD3+ T cells in the CT	1.273 (0.832, 1.948)	**0.267**			1.432 (0.856, 2.398)	**0.172**		
CD8+/CD3+ T cells in the IM	1.193 (0.781, 1825)	**0.414**			1.240 (0.745, 2.064)	**0.409**		
CD8+/CD3+ T cells in the microenvironment	1.315 (0.860, 2.010)	**0.207**	1.513 (0.946, 2.421)	**0.084**	1.874 (1.108, 3.168)	**0.019**	1.743 (0.984, 3.088)	**0.057**
Immunoscore	0.861 (0.554, 1.340)	**0.508**			1.027 (0.611, 1.726)	**0.921**		

Bold values indicate P < 0.05; tumor location: Left side or right side; CI, Confidence interval; CT, Core of the tumor; HR, Hazard ratio; IM, Invasive margin; PFS, Progression-free survival; OS, Overall survival.

### Predictive and Prognostic Value of Immune Cell Infiltration for Patients on Chemotherapy Regimens

**Figure 2:**
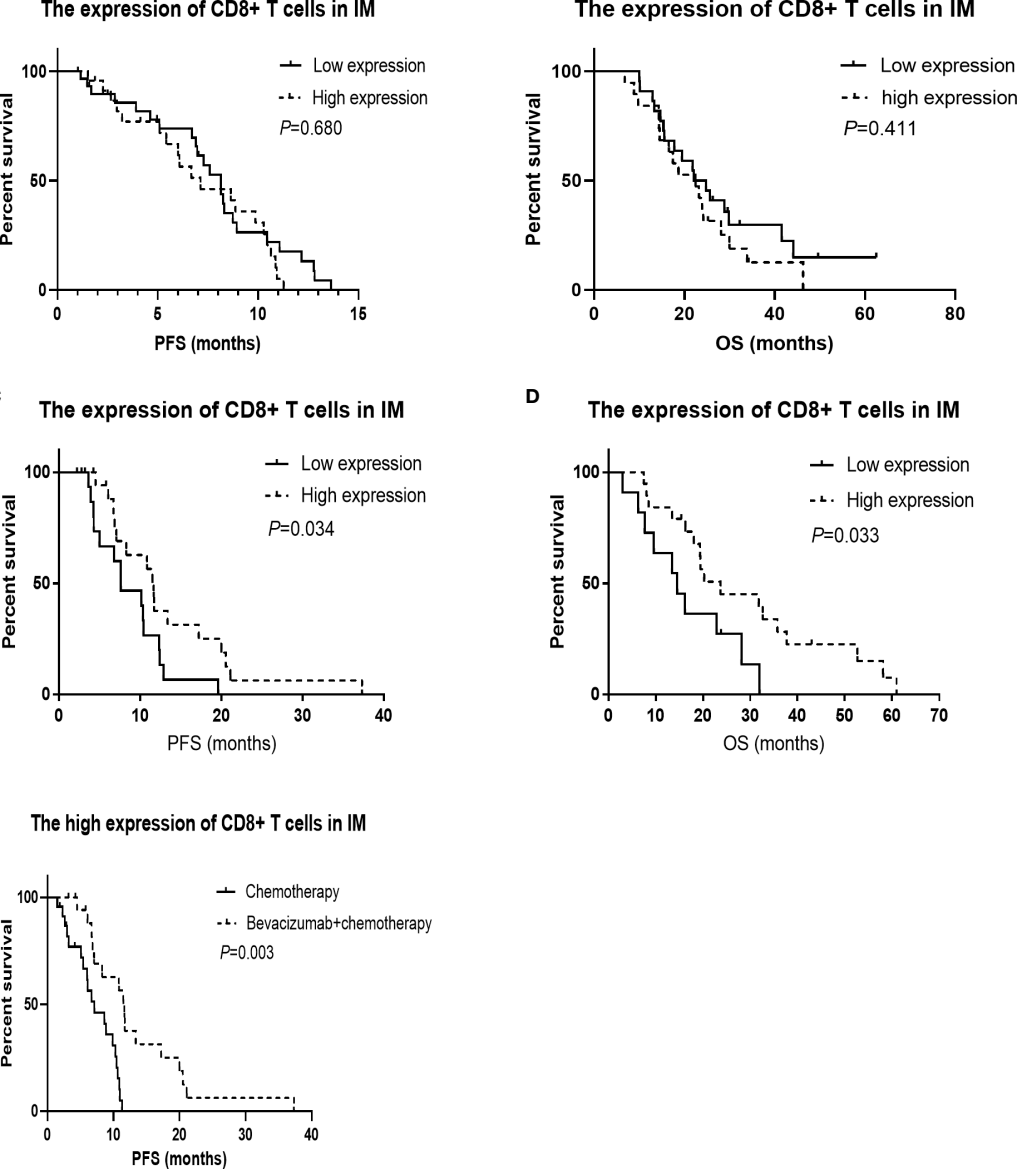
Kaplan-Meier survival curves according to the tumor infiltrated immune cells. CD8+ in invasive margin (IM) predicts the effect of bevacizumab plus chemotherapy, and patients with high CD8+ in IM have longer overall survival (OS) treated with bevacizumab plus chemotherapy. **(A)** The PFS was not significantly worse in the low CD8+ T cells in IM group treated with chemotherapy (*P* = 0.680). **(B)** The OS was not significantly worse in the low CD8+ T cells in IM group treated with chemotherapy (*P* = 0.411). **(C)** The progression-free survival (PFS) was significantly worse in the low CD8+ T cells in IM group treated with chemotherapy combined with bevacizumab (*P* = 0.034). **(D)** The OS was significantly worse in the low CD8+ T cells in IM group treated with chemotherapy combined with bevacizumab (*P* = 0.033). **(E)** For the patients with high CD8+ T cells in IM group, The PFS was significantly better in the arm of chemotherapy combined with bevacizumab than that of chemotherapy alone (*P* = 0.003).

For the 55 patients who received chemotherapy alone as the first-line treatment, the median PFS and OS were 7.59 mo and 22.47 mo, respectively. As shown in **Table 4**, although there was no association between CD3 or CD8 expression levels in the tumor microenvironment and the overall response rate (ORR) or disease control rate (DCR), the ORR was higher for patients with a higher Immunoscore (40.0% vs. 60.0%, *P* = 0.022). A lower CD8+/CD3+ T cell ratio in the microenvironment was associated with a significantly longer PFS (8.64 mo vs. 6.01 mo, *P* = 0.017), and patients with more CD3+ T cells in the CT had a longer OS (16.56 mo vs. 25.66 mo, *P* = 0.029). The CD8+ T cells in the IM did not show any association with PFS (8.15 mo vs. 7.13 mo, *P* = 0.680, **Figure 2A**) and OS (23.44 mo vs. 22.47 mo, *P* = 0.411, **Figure 2B**). In addition, the Cox analysis presented in **Table 5** shows that pathological grade (HR 2.450, 95% CI 1.021–5.665, *P* = 0.045) and the CD8+ to CD3+ T cell ratio in the microenvironment (HR 2.863, 95% CI 1.405–5.833, *P* = 0.004) were associated with PFS, but none of the evaluated factors influenced OS.

**Table 4:** Relationships of immune cell infiltration with efficacy for patients on chemotherapy regimens.

Factors	Expression	N	ORR		DCR		PFS		OS	
			N (%)	*P*	N (%)	*P*	Months	*P*	Months	*P*
CD3+ T cells in the CT	Low	28	10 (37.5%)	**1.000**	24 (85.7%)	**1.000**	6.90	**0.111**	16.56	**0.029**
	High	25	8 (32.0%)		22 (88.0%)		8.64		25.66	
	NA	2								
CD3+ T cells in the IM	Low	26	6 (23.1%)	**0.148**	22 (84.6%)	**0.704**	6.67	**0.057**	17.74	**0.481**
	High	27	12 (44.4%)		24 (88.9%)		8.31		23.92	
	NA	2								
CD8+ T cells in the CT	Low	24	9 (37.5%)	**0.148**	21 (87.5%)	**1.000**	8.25	**0.477**	25.66	**0.224**
	High	29	9 (31.0%)		25 (86.2%)		6.67		18.69	
	NA	2								
CD8+ T cells in the IM	Low	30	9 (30.0%)	**0.565**	26 (86.7%)	**1.000**	8.15	**0.341**	22.11	**0.299**
	High	23	9 (39.1%)		20 (87.0%)		7.13		22.47	
	NA	2								
CD8+/CD3+ T cells in the CT	Low	24	8 (33.3%)	**1.000**	22 (91.7%)	**0.436**	7.13	**0.452**	24.77	**0.198**
	High	29	10 (34.5%)		24 (82.8%)		8.15		18.69	
	NA	2								
CD8+/CD3+ T cells in the IM	Low	24	11 (40.7%)	**1.000**	24 (88.9%)	**0.704**	8.15	**0.088**	22.11	**0.841**
	High	29	7 (26.9%)		22 (84.6%)		6.67		23.19	
	NA	2								
CD8+/CD3+ T cells in the microenvironment	Low	27	11 (40.7%)	**0.387**	25 (92.6%)	**0.250**	8.64	**0.017**	25.66	**0.055**
	High	26	7 (26.9%)		21 (80.8%)		6.01		17.74	
	NA	2								
Immunoscore	Low	38	9 (23.7%)	**0.022**	32 (84.2%)	**0.658**	7.13	**0.298**	21.82	**0.689**
	High	15	9 (60.0%)		14 (93.3%)		8.64		22.47	
	NA	2								

Bold values indicate P < 0.05; CT, Core of the tumor; DCR, Disease control rate; IM, Invasive margin; PFS, Progression-free survival; NA, Not applicable; ORR, Overall response rate; OS, Overall survival.

**Table 5:** Univariate and multivariate analyses of immune cell infiltration and clinicopathological factors on survival for patients on chemotherapy regimens.

	PFS				0S			
	Univariate analysis		Multivariate analysis		Univariate analysis		Multivariate analysis	
	HR (95% CI)	*P*	HR (95% CI)	*P*	HR (95% CI)	*P*	HR (95% CI)	*P*
Age	0.982 (0.531, 1.816)	**0.954**			1.022 (0.512, 2.042)	**0.951**		
Tumor location	1.050 (0.543, 2.029)	**0.885**			1.483 (0.642, 3.427)	**0.356**		
Pathological grade	1.197 (0.635, 2.256)	**0.579**	2.405 (1.021, 5.665)	**0.045**	1.072 (0.529, 2.171)	**0.847**		
T stage	0.793 (0.404, 1.557)	**0.500**	0.460 (0.190, 1.114)	**0.085**	1.194 (0.590, 2.417)	**0.622**		
N stage	1.185 (0.557, 2.518)	**0.659**			1.326 (0.538, 3.268)	**0.540**		
Synchronous/metachronous metastasis	0.424 (0.182,0.986)	**0.046**			0.420 (0.174, 1.017)	**0.054**	0.449 (0.179, 1.127)	**0.088**
CD3+ T cells in the CT	0.610 (0.330, 1.130)	**0.116**			0.521 (0.262, 1.035)	**0.063**	0.498 (0.223, 1.067)	**0.073**
CD3+ T cells in the IM	0.554 (0.298, 1.029)	**0.062**			0.882 (0.445, 1.748)	**0.719**		
CD8+ T cells in the CT	1.244 (0.679, 2.280)	**0.479**			1.400 (0.707, 2.771)	**0.334**		
CD8+ T cells in the IM	1.352 (0.722, 2.535)	**0.345**			1.429 (0.726, 2.816)	**0.302**		
CD8+/CD3+ T cells in the CT	1.362 (0.741, 2.505)	**0.320**			1.371(0.695, 2.707)	**0.363**		
CD8+/CD3+ T cells in the IM	1.726 (0.913, 3.264)	**0.093**			0.982 (0.499, 1.933)	**0.959**		
CD8+/CD3+ T cells in the microenvironment	2.125 (1.128, 4.002)	**0.020**	2.863 (1.405, 5.833)	**0.004**	1.738 (0.877, 3.445)	**0.114**		
Immunoscore	0.707 (0.366, 1.366)	**0.302**			1.169 (0.544, 2.514)	**0.690**		

Bold values indicate P < 0.05; tumor location: Left side or right side; CI, Confidence interval; CT, Core of the tumor; HR, Hazard ratio; IM, Invasive margin; PFS, Progression-free survival; OS, Overall survival

### Predictive and Prognostic Value of Immune Cell Infiltration for Patients on Bevacizumab Plus Chemotherapy

The median PFS and OS of the 38 patients who received bevacizumab plus chemotherapy as their first-line regimen were 10.31 mo and 19.45 mo, respectively. There was no association between immune cell infiltration in the tumor microenvironment and ORR or DCR. Patients with a greater population of CD8+ T cells in the IM had a longer PFS (7.62 mo vs. 11.66 mo, *P* = 0.034, **Figure 2C**) and OS (14.55 mo vs. 23.72 mo, *P* = 0.033, **Figure 2D**), as shown in **Table 6**. According to the Cox analysis, a higher percentage of CD8+ T cells in the IM indicated a 57.1% increase in PFS (HR 0.429, 95% CI 0.189–0.973, *P* = 0.043) and a 60.6% increase in OS (HR 0.394, 95% CI 0.162–0.963, *P* = 0.041) compared to a lower percentage, as shown in **Table 7**. What is more, the patients with high CD8+ T cells in IM group, The PFS was significantly better in the arm of chemotherapy combined with bevacizumab than that of chemotherapy alone (7.13 mo vs. 11.66 mo, *P* = 0.003, **Figure 2E**).

**Table 6:** Relationships of immune cell infiltration with efficacy for patients on bevacizumab plus chemotherapy.

Factors	Expression	N	ORR		DCR		PFS		OS	
			N (%)	*P*	N (%)	*P*	Months	*P*	Months	*P*
CD3+ T cells in the CT	Low	16	4 (25.0%)	**1.000**	16 (100.0%)	**1.000**	10.32	**0.579**	19.45	**0.396**
	High	19	5 (26.3%)		18 (94.7%)		8.28		19.38	
	NA	3								
CD3+ T cells in the IM	Low	18	6 (33.3%)	**0.443**	18 (100.0%)	**0.486**	10.32	**0.676**	19.45	**0.330**
	High	17	3 (17.6%)		16 (0.941)		8.28		19.38	
	NA	3								
CD8+ T cells in the CT	Low	20	3 (15.0%)	**0.245**	19 (95.0%)	**1.000**	8.28	**0.261**	19.38	**0.536**
	High	16	6 (37.5%)		16 (100.0%)		11.50		22.90	
	NA	2								
CD8+ T cells in the IM	Low	17	3 (17.6%)	**0.451**	16 (94.1%)	**0.472**	7.62	**0.034**	14.55	**0.033**
	High	19	6 (31.6%)		19 (100.0%)		11.66		23.72	
	NA	2								
CD8+/CD3+ T cells in the CT	Low	18	4 (22.2%)	**0.711**	17 (94.4%)	**1.000**	8.28	**0.725**	28.12	**0.233**
	High	17	5 (29.4%)		17 (100.0%)		10.32		16.26	
	NA	3								
CD8+/CD3+ T cells in the IM	Low	19	4 (21.1%)	**0.700**	18 (94.7%)	**1.000**	8.28	**0.448**	19.38	**0.342**
	High	16	5 (31.3%)		16 (100.0%)		11.66		19.45	
	NA	3								
CD8+/CD3+ T cells in the microenvironment	Low	17	4 (23.5%)	**1.000**	16 (94.1%)	**0.486**	8.28	**0.725**	28.12	**0.120**
	High	18	5 (27.8%)		18 (100.0%)		10.32		16.26	
	NA	3								
Immunoscore	Low	21	5 (23.8%)	**1.000**	20 (95.2%)	**1.000**	10.32	**0.874**	20.24	**0.526**
	High	15	4 (26.7%)		15 (100.0%)		10.38		19.38	
	NA	2								

Bold values indicate P < 0.05; CT, Core of the tumor; DCR, Disease control rate; IM, Invasive margin; PFS, Progression-free survival; NA, Not applicable; ORR, Overall response rate; OS, Overall survival.

**Table 7:** Univariate and multivariate analyses of immune cell infiltration and clinicopathological factors on survival for patients on bevacizumab plus chemotherapy.

	PFS				0S			
	Univariate analysis		Multivariate analysis		Univariate analysis		Multivariate analysis	
	HR (95% CI)	*P*	HR (95% CI)	*P*	HR (95% CI)	*P*	HR (95% CI)	*P*
Age	0.246 (0.032, 1.888)	**0.178**			0.413 (0.096, 1.786)	**0.413**		
Tumor location	1.034 (0.466, 2.294)	**0.934**			0.370 (0.302, 1.562)	**0.370**		
Pathological grade	1.345 (0.637, 2.838)	**0.436**			1.771 (0.796, 3.942)	**0.161**		
T stage	0.830 (0.388, 1.777)	**0.632**			1.149 (0.490, 2.693)	**0.749**		
N stage	0.551 (0.209, 1.449)	**0.227**			0.645 (0.213, 1.951)	**0.437**		
Synchronous/metachronous metastasis	0.883 (0.397, 1.963)	**0.761**			0.407 (0.175, 0.949)	**0.037**		
CD3+ T cells in the CT	1.233 (0.587, 2.593)	**0.589**			0.696 (0.300, 1.614)	**0.398**		
CD3+ T cells in the IM	1.170 (0.561, 2.440)	**0.676**			0.661 (0.285, 1.531)	**0.333**		
CD8+ T cells in the CT	0.662 (0.321, 1.365)	**0.264**			0.781 (0.357, 1.709)	**0.537**		
CD8+ T cells in the IM	0.441 (0.203, 0.957)	**0.038**	0.429 (0.189, 0.974)	**0.043**	0.401 (0.168, 0.956)	******0.039******	0.394 (0.162, 0.963)	**0.041**
CD8+/CD3+ T cells in the CT	0.876 (0.419, 1.831)	**0.725**			1.680 (0.709, 3.982)	**0.238**		
CD8+/CD3+ T cells in the IM	0.748 (0352, 1.588)	**0.450**			1.498 (0.647, 3.473)	**0.346**		
CD8+/CD3+ T cells in the microenvironment	0.876 (0.419, 1.831)	**0.725**			2.004 (0.820, 4.901)	**0.127**		
Immunoscore	1.060 (0.515, 2.181)	**0.874**			0.765 (0.333, 1.758)	**0.527**		

Bold values indicate P < 0.05; tumor location, Left side or right side; CI, Confidence interval; CT, Core of the tumor; HR, Hazard ratio; IM, Invasive margin; PFS, Progression-free survival; OS, Overall survival.

## Discussion

Our previous report on CRC showed that a high number of immune cells in the tumor microenvironment is associated with some positive predictors of clinical characteristics and blood indexes (16). Herein, we examined the levels of infiltrating CD3+ and CD8+ T cells in primary cancer tissues from mCRC patients who received chemotherapy alone or in combination with bevacizumab. We found that older patients had fewer CD3+ T cells in both the CT and IM, lower Immunoscores, and higher CD8+ to CD3+ T cell ratios in the IM and the total microenvironment. CD8+ expression in the IM was lower in patients with N0 stage disease. Patients with synchronous metastasis had a lower percentage of CD3+ T cells in both the CT and IM and a higher ratio of CD8+ to CD3+ T cells in the CT, IM, and total microenvironment. Synchronous/metachronous metastasis and the CD8+ to CD3+ T cell ratio in the microenvironment were independent prognostic factors for OS regardless of treatment regimen. In the chemotherapy only group, patients with a high Immunoscore had a high ORR, those with a low CD8+/CD3+ T cell ratio in the microenvironment had a significantly longer PFS, and those with more CD3+ T cells in the CT had a longer OS. In the chemotherapy combined with bevacizumab group, patients with a higher percentage of CD8+ T cells in the IM had a longer PFS and OS. The results from this study suggest that the TIL status in primary cancer samples is a feasible predictor of therapeutic response in mCRC. The most interesting finding is that according to multivariate Cox analysis, none of the factors influenced OS in patients on chemotherapy alone, while a higher percentage of CD8+ T cells in the IM increased OS in patients treated with bevacizumab and chemotherapy.

As we discovered before in the CRC group containing patients at all clinical stages, clinicopathologic characteristics may reflect immune cell concentrations in the primary tumor microenvironment (16). In this study on mCRC, patients’ clinicopathologic characteristics, including age, N stage, and synchronous/metachronous metastasis, were associated with the level of immune cell infiltration in primary tumors. This finding can be explained by the hypothesis that a superior host immune response may limit the progression and invasion of malignancies; thus, patients with a larger number of immune cells in the microenvironment may have a better prognosis. Aging results in declining health and an increased risk of cancer, in which decreased immune system activity is thought to play a key role (17); moreover, the tumor immuno-microenvironment is altered as a result of age-related immune dysfunction (18). This is consistent with the findings of our previous (16) and present studies, namely, that age is associated with immune cell concentrations in disease. Studies have confirmed the strong association between CD8+ T cells and metastasis, and patients with more distant metastases have a significantly lower density of lymphocytes in tumors (19). The present study also found that N stage and synchronous/metachronous metastasis were associated with immune cell concentrations in mCRC. Though it shows some differences between these articles, we assume the explanation that due to patients with different TNM stage.

The identification of reliable prognostic factors for CRC is the focus of intensive clinical and translational research. In this study, we observed that the ratio of CD8+ to CD3+ T cells in the microenvironment was a negative prognostic factor for OS, regardless of treatment regimen. For patients treated with chemotherapy, the CD8+/CD3+ T cell ratio in the microenvironment was a negative prognostic factor for PFS, and CD3+ T cells in the CT were a positive prognostic factor for OS. Since the early 1900s, tumor immune infiltration has been suspected to be a positive factor for patient prognosis (20). Tumor cells interact with the microenvironment and are influenced by signals from stromal, endothelial, inflammatory, and immune cells (21). Tumors are often infiltrated by various populations of lymphocytes, macrophages or mast cells. The presence of a high number of lymphocytes, especially T cells, has been reported to be an indicator of good prognosis for patients with CRC (22). Due to the uneven infiltration of T cell in colon tumors, attention has been focused on the predictive value of T cells in the CT and IM. The Immunoscore, a derived immune score, summarizes the expression of CD8+ and CD3+ T cell within the CT and IM. The Immunoscore has been confirmed to predict clinical prognosis in patients with early- (12) and advanced-stage (23) CRC. We also found that patients with a high Immunoscore may have a high ORR when treated with chemotherapy. Interestingly, we found that the ratio of CD8+ to CD3+ T cells in the microenvironment was a negative prognostic factor for OS, regardless of treatment regimen, and it was also a negative prognostic factor for PFS for patients treated with chemotherapy. We assume that the ratio of each T-cell subtype should be in a suitable range. Elucidation of the mechanisms active within the T-cell network is complicated by the complex associations between the various T-cell subtypes and cytokines. These results proved that the local immune context, including the density, phenotype, activation status, and localization of immune cells, is a potential prognostic factor for ORR, PFS and OS in mCRC and implied that immunological criteria should be of interest in clinical practice and added to tumor staging to improve patient outcomes. We did not find relationship between MS status and the expression of immune cell, the potential reason as follows: firstly, the population with MSI was so small; secondly, the newest research in Nov 2020 reported that different immune checkpoint inhibitors may be beneficial for selected CRC patients irrespective of MSI status, because the subtype of immune cells will more directly affect the efficacy of immunotherapy (24). In addition, particular attention should be given to the analysis of the lymphocytic infiltrate in tumors before treatment with chemotherapy and bevacizumab or immunotherapies.

Bevacizumab, a humanized IgG1 mAb against VEGF-A and inhibiting angiogenesis (25), plus chemotherapy as first-line treatment for patients with mCRC provided a longer median PFS and OS (26). The use of Bevacizumab may modulate tumor microenvironment and synergize with immunotherapy. New immunotherapy approaches for mCRC with MSI-High are clearly warranted. However, very few responses have been observed in non-MSI-High mCRC patients treated with PD1/PDL1 checkpoint inhibitors (27). Based on the 95% mCRC patients are MSI-L type, improvement of their benefits in immunotherapy is a critical problem. Interestingly, preclinical studies have shown the formation of tumor derived blood and lymphatic vascular promote an immunosuppressive microenvironment by modulating the recruitment, adhesion, trafficking, and function of immune cells (28). The combination of checkpoint inhibitors and the anti-VEGF mAb bevacizumab may be beneficial.

We found that CD8+ T cells in the IM had significant prognostic value for both PFS and OS in patients treated with bevacizumab plus chemotherapy. CD8+ T cells are cytotoxic T lymphocytes that directly attack cancer cells and play a central role in anticancer immunity (29). A previous study reported substantial evidence that the CD8+ T cell density was associated with long-term survival in various types of cancer (12, 30). Furthermore, the CD8+ T cell density was reported to be associated with the therapeutic efficacy of chemotherapy and radiotherapy (31, 32). In addition, marked CD8+ T cell infiltration was observed in MSI patients, and mismatch repair deficiency created multiple immunogenic peptides that became stimuli and targets of antitumor immune responses (33). The most interesting finding is that the multivariate analysis found no effect of CD3+ or CD8+ T cells or the Immunoscore on OS in patients treated with chemotherapy alone, while a higher percentage of CD8+ T cells in the IM increased OS in patients treated with bevacizumab and chemotherapy. These data might indicate that cytotoxic T lymphocytes play a greater role in bevacizumab-based regimens than in those without bevacizumab. Antiangiogenic treatment can normalize of the tumor vasculature (34). The vascular endothelium has a barrier function and plays a role in the activation of immunity by increasing endothelial cell adhesion molecules which could interact with macrophages, NK cells, T cells, and B cells for antigen recognition during the immune response. And this therapeutic may change how the tumor microenvironment establishes complex networks to escape immune attacks when antitumor cytotoxic T cell activity is perturbed by the downregulation of stimulatory signals, the upregulation of inhibitory signals or both (35). In addition, VEGF can increase PD-1 expression on T cells and promote the infiltration and activation of CD8+ T cells (36). These results may support the hypothesis that bevacizumab might have an immune-enhancing effect, including ADCC activity, following the accumulation of CD8+ T cells in the tumor microenvironment. Recent studies have reported that the presence of CD8+ T cells in a tumor is a positive biomarker for anti-PD1 therapy (37). Clinical data also support a potentially synergistic interaction between antiangiogenic treatment and immunotherapy. The combination of anti-VEGF treatment and immunotherapy has produced surprisingly significant results in hepatocellular carcinoma and non-small cell lung cancer (38, 39). The combination regimen of immunotherapeutics plus bevacizumab and chemotherapy might be a potential approach to improve the limited efficacy of immunotherapy, especially in mCRC patients with high CD8 expression in the tumor microenvironment.

There are limitations and possible biases in this study. The sample size was not large enough, and because of the nature of retrospective studies, it is possible that patient selection was biased according to sample availability. Additionally, we did not analyze specific subtypes of T cells other than CD3+ and CD8+ immune cells; these subtypes may have different roles in the tumor microenvironment and indicate diverse prognoses. Regardless, our study tested the hypothesis that tumor-infiltrating immune cells predict treatment efficacy, and the results provide new evidence for the combination of immunotherapy with chemotherapy in mCRC, which has not been previously reported.

## Conclusions

Our study confirmed that CD3+ and CD8+ immune cells can be used to predict treatment outcome and the prognosis of mCRC patients treated with chemotherapy with or without bevacizumab. Furthermore, patients with a large population of CD8+ T cells will have better treatment outcomes with the combination of chemotherapy plus bevacizumab, while the expression of CD3 and the CD8+/CD3+ T cell ratio are predictors of the effect of chemotherapy. In the future, it will be exciting to explore the potential of TILs as promising biomarkers that may also guide therapeutic decisions, especially in the times of immunotherapy.

## Data Availability Statement

The original contributions presented in the study are included in the article/supplementary material. Further inquiries can be directed to the corresponding authors.

## Ethics Statement

The studies involving human participants were reviewed and approved by Human Ethics Approval Committee at Sun Yatsen University Cancer Center. The patients/participants provided their written informed consent to participate in this study. Written informed consent was obtained from the individual(s) for the publication of any potentially identifiable images or data included in this article.

## Author Contributions

GG designed research. YW, JD, and QQ analyzed the data and wrote the paper. SL collected data. XCh contributed new reagents and analytic tools. XCa and HQ performed experiment research. BZ amended paper. All authors contributed to the article and approved the submitted version.

## Funding

The present study was supported by the National Natural Science Foundation of China (grant No.813002141) and the Guangdong Provincial Natural Science Foundation (grant No. 2017A030313685).

## Conflict of Interest

The authors declare that the research was conducted in the absence of any commercial or financial relationships that could be construed as a potential conflict of interest.
